# Possible Neuropathology of Sleep Disturbance Linking to Alzheimer’s Disease: Astrocytic and Microglial Roles

**DOI:** 10.3389/fncel.2022.875138

**Published:** 2022-06-09

**Authors:** Shu-Yun Xiao, Yi-Jie Liu, Wang Lu, Zhong-Wei Sha, Che Xu, Zhi-Hua Yu, Shin-Da Lee

**Affiliations:** ^1^Department of Mental Diseases, Shanghai Municipal Hospital of Traditional Chinese Medicine, Shanghai University of Traditional Chinese Medicine, Shanghai, China; ^2^School of Rehabilitation Science, Shanghai University of Traditional Chinese Medicine, Shanghai, China; ^3^Institute of Rehabilitation Science, Shanghai University of Traditional Chinese Medicine, Shanghai, China; ^4^Department of Traditional Treatment, Longhua Hospital, Shanghai University of Traditional Chinese Medicine, Shanghai, China; ^5^School of Basic Medicine, Shanghai University of Traditional Chinese Medicine, Shanghai, China; ^6^Shanghai Geriatric Institute of Chinese Medicine, Shanghai University of Traditional Chinese Medicine, Shanghai, China; ^7^Department of Physical Therapy, Graduate Institute of Rehabilitation Science, China Medical University, Taichung, Taiwan; ^8^Department of Physical Therapy, Asia University, Taichung, Taiwan

**Keywords:** sleep disturbance, Alzheimer’s disease, astrocyte, microglia, amyloid beta

## Abstract

Sleep disturbances not only deteriorate Alzheimer’s disease (AD) progress by affecting cognitive states but also accelerate the neuropathological changes of AD. Astrocytes and microglia are the principal players in the regulation of both sleep and AD. We proposed that possible astrocyte-mediated and microglia-mediated neuropathological changes of sleep disturbances linked to AD, such as astrocytic adenosinergic A1, A2, and A3 regulation; astrocytic dopamine and serotonin; astrocyte-mediated proinflammatory status (TNFα); sleep disturbance-attenuated microglial CX3CR1 and P2Y12; microglial Iba-1 and astrocytic glial fibrillary acidic protein (GFAP); and microglia-mediated proinflammatory status (IL-1b, IL-6, IL-10, and TNFα). Furthermore, astrocytic and microglial amyloid beta (Aβ) and tau in AD were reviewed, such as astrocytic Aβ interaction in AD; astrocyte-mediated proinflammation in AD; astrocytic interaction with Aβ in the central nervous system (CNS); astrocytic apolipoprotein E (ApoE)-induced Aβ clearance in AD, as well as microglial Aβ clearance and aggregation in AD; proinflammation-induced microglial Aβ aggregation in AD; microglial-accumulated tau in AD; and microglial ApoE and TREM2 in AD. We reviewed astrocytic and microglial roles in AD and sleep, such as astrocyte/microglial-mediated proinflammation in AD and sleep; astrocytic ApoE in sleep and AD; and accumulated Aβ-triggered synaptic abnormalities in sleep disturbance. This review will provide a possible astrocytic and microglial mechanism of sleep disturbance linked to AD.

## Introduction to Sleep Disturbance and Alzheimer’s Disease

Sleep plays an important role in maintaining normal biological and physiological functions. Disturbed sleeping affects not only the health condition but also the life quality of individuals. Chronic sleep loss is linked to a wide range of unhealthy conditions, such as altered food intake, weight loss or gain, skin lesions, compromised thermoregulation, and even death, causing mental and economic burdens to the family (Rechtschaffen et al., 1989; Siegel, 2008). Sleep states can be characterized by electroencephalogram (EEG) activity, non-rapid eye movement (NREM), and rapid eye movement (REM) sleep. NREM sleep is featured with slow-wave EEG, while the REM sleep stage shows higher frequency brain activity with low amplitude but high-frequency EEG (Scammell et al., 2017). The timing for sleep is controlled by sleep and circadian rhythm. The former is regulated by homeostasis, which determines the sleep duration and amount, and the latter relies on the function of the suprachiasmatic nucleus of the lateral hypothalamus (LH) (Partch et al., 2014; Borbély et al., 2016).

Sleeping disturbance can be triggered by the disturbance of environmental and physiological factors (Donlea and Shaw, 2009) including senescence, genetic mutation, and disease states (Potter et al., 2016; Charrier et al., 2017; Veatch et al., 2017). Sleep gene mutation is seen in the disruption of synaptic activity and neuronal homeostasis, which may contribute to the sleeping disturbance in multiple psychiatric disorders and diseases (Potter et al., 2016; Mulas et al., 2019). Specifically, sleep disturbance in neurodegenerative diseases, for example, Alzheimer’s disease (AD), has gained increasingly wide attention worldwide. Patients with AD have been suffering from different magnitudes of sleeping difficulties, which is also a classical symptom of AD (McCleery and Sharpley, 2020; Matsumoto and Tsunematsu, 2021).

Sleep disturbance and AD have huge impacts on individuals and society, therefore growing to be global health concerns. Loss of sleep accelerates AD progression not only by emotionally affecting the mental states of patients but also through the pathological changes of AD (Irwin and Vitiello, 2019; Van Egroo et al., 2019; Wang and Holtzman, 2020). Sleep disorders and AD partially share pathological mechanisms and induce similar cognitive deficits (Uddin et al., 2020; Lucey et al., 2021). Therefore, it is important to further characterize the possible neuropathological changes of sleep disturbance linked to AD. AD is well acknowledged that genetic disturbance is an important pathological driving force of AD (Lane et al., 2018). Poor sleep seems to exacerbate neurodegeneration (Grimmer et al., 2020; Baril et al., 2022; Blackman et al., 2022). Genome-wide studies identified more than 20 high-risk genes associated with AD from thousands of patients. Mutation of a few typical genes, including amyloid precursor protein and apolipoprotein (APOE), accounts for a majority of AD development (Bateman et al., 2011; Lane et al., 2018). It is known that the fibrillar conformation of amyloids’ oligomeric forms interacts with the innate immune system to initiate a transcriptional inflammatory response (Lee et al., 2020). Mutation of these genes leads to amyloid beta (Aβ) abnormal protein functions, which can trigger a series of immune reactions in the central nervous system (CNS). In patients without risk alleles, chronic sleep restriction induces frontal cortical mitochondrial dysfunction and mitochondria-related Aβ accumulation, which is considered to be a risk factor for the pathophysiology of sporadic Alzheimer’s disease (Zhao et al., 2016, 2019; Liu et al., 2020). Misfolded Aβ and tau trigger glial cell vigilance and then induce a series of complex immune responses with neuroinflammation and neurodegeneration (Wu et al., 2021). Thus, neuro-immune interaction has surged as a critical convergent point to study the pathological mechanism of AD.

Sleep is essential for the recharging of immune system, and sleep disturbance-induced immune dysfunction could promote AD psychosis (Wu et al., 2019). It has been reported that sleep disturbance can trigger the proinflammatory process of the body to protect itself from harm during daytime.

## Astrocytic and Microglial Roles in Sleep or Sleep Disturbance

Central nervous system infections gained more and more attention in the exploration of the pathology of sleep homeostasis (Tesoriero et al., 2019). Accumulating body of research indicates the principal role of astrocytes and microglia in the regulation of both sleep and AD (Garofalo et al., 2020; Gentry et al., 2022). Chronic sleep deprivation was found to increase the activity of both astrocyte and microglia (Bellesi et al., 2017), which are distributed widely across the CNS, are in charge of controlling neuroinflammation, and have surged to be the major players in the regulation of sleep-awake cycle.

### Astrocyte in Sleep or Sleep Disturbance

Circadian oscillation and sleep homeostasis determine the sleep cycle (Fuller et al., 2006). Astrocytes have been found to participate in the regulation of sleep cycle and homeostasis (Brown et al., 2012; Czeisler et al., 2019; Lazarus et al., 2019; Bojarskaite et al., 2020). Studies monitoring astrocytic Ca^2+^ dynamics *in vivo* showed that astrocytic Ca^2+^ activity was characterized by a spatial-temporal feature corresponding to sleep-wake cycles (Bojarskaite et al., 2020; Peng et al., 2020). Astrocytes show lower astrocytic Ca^2+^ activity during the slow-wave sleep state compared with that in the awake and NREM state. While increased astrocytic Ca^2+^ signaling had been detected before the transition from slow-wave sleep to wakefulness (Bojarskaite et al., 2020; Vaidyanathan et al., 2021). It is speculated that the downregulation of astrocytic activity is accommodated by the relative inactive state of neural activity during sleep (Bojarskaite et al., 2020). Interestingly, this study also discovers that the astrocytic process presents more frequent Ca^2+^ signals compared with the glial cell body during a slow-wave sleep state (Bojarskaite et al., 2020). Astrocytes use the process to scan the perturbation of synaptic activity. Therefore, although the general activity of astrocytes is low, Ca^2+^ activity in the process could preserve their ability in maintaining the homeostasis of CNS during sleep.

### Astrocytic A1, A2, and A3 Regulation in Sleep

How do astrocytes or astrocytic processes accomplish this process? Astrocyte exerts its actions mainly through the release of adenosine (Porkka-Heiskanen et al., 1997; Halassa et al., 2009), which is a metabolizing production of adenosine triphosphate (ATP). Once released into the extracellular space, it binds with A1, A2 (A2A and A2B subtypes), and A3 receptors (A3Rs), which are all G-protein-coupled receptors on the neural membrane (Fredholm et al., 1994). A1 and A3A Rs mainly inhibit the concentration of cyclic adenosine monophosphate (cAMP) and downregulate neural activity. In contrast, A2A and A2B Rs activate the cAMP intracellular signaling pathway to upregulate neural activity. Imbalanced activation of these two groups of receptors could cause perturbation in the homeostasis of the neural network associated with sleep disorder and cognitive deficits (Portas et al., 1997; Pereira et al., 2005). Few reviews have summarized the specific role of adenosine, A1 and A2Rs, in the regulation of sleep and wakefulness (Huang et al., 2014; Lazarus et al., 2019). In the model proposed by Lazarus and his colleagues, these two types of receptors are phase-locked with a certain period of sleep (Lazarus et al., 2019). A1Rs are mostly located on presynapses and are responsible for maintaining the slow-wave oscillation during the sleep state by reducing the presynaptic release of neurotransmitters. It has been found that the administration of A1R antagonist in a variety of brain regions facilitates the sleep process (Rainnie et al., 1994; Liu and Gao, 2007; Oishi et al., 2008), while A_2A_Rs allows the brain to enter a sleep state. The activation of A2aR in these brain regions promotes REM (Satoh et al., 1996; Urade et al., 2003) and the transition from sleep to awake state. However, A2aR activation-induced waking is often accompanied by higher-order cognitive deficits resembling the consequence of sleep loss (Urry and Landolt, 2015). Therefore, hyperactivation of A2aR might underlie the stimulant-induced impairment of attention.

### Astrocytic Dopamine and Serotonin in Sleep

Astrocytes also mediate sleep through dopamine and serotonin. Neural circuits transmitting monoamine signals help maintain the sleep homeostasis. Glial cells are known to metabolize monoamine (Nall and Sehgal, 2014). In turn, norepinephrine can control the network formation between astrocyte and neurons (Bar El et al., 2019). Studies in drosophila revealed that dopamine promoted awake state and facilitated memory formation during sleep. Another monoamine family member, serotonin, also enhanced the sleeping process through different signaling pathways though (for review see Nall and Sehgal (2014)). AANAT1, an astrocyte gene that acetylates and inactivates monoamine, is found critical for the regulation of serotonin and dopamine levels in the brain. Studies have shown that AANAT1 can acetylate dopamine and affect melatonin production to regulate sleep (Cheng et al., 2012; Kulczykowska et al., 2017; Fagan et al., 2021), and it can limit the accumulation of serotonin and dopamine in the brain after sleep deprivation (Nall and Sehgal, 2014; Davla et al., 2020). Sleep deprivation leads to a significant increase in serotonin and dopamine, and this effect is reversed by astrocyte, but not neuron-specific AANAT1 mutation flies (Davla et al., 2020), suggesting that astrocyte-mediated monoamine metabolism is important for sleep homeostasis.

### Astrocyte-Mediated Proinflammatory Status in Sleep

Apart from the astrocyte-neuron interaction through Ca^2+^ signal modulation, the astrocyte-mediated inflammatory pathway is another leading player in the regulation of sleep. One of the important regulatory substances of sleep associated with astrocyte is tumor necrosis factor alpha (TNFα), a proinflammatory factor. TNF-α not only promotes non-REM but also contributes to the establishment of sleep homeostasis following sleep deprivation (Yamasu et al., 1992). This function is mainly accomplished through the regulation of neural activity and synaptic plasticity of sleep circuits (Turrin and Rivest, 2004; Kaneko et al., 2008). A recent study discovered that knockdown Drosophila TNF-α homolog, Eiger, specifically in astrocyte could largely reduce the sleep duration (Vanderheyden et al., 2018a), indicating a waking-favor role the TNF-α plays in sleep homeostasis. In fact, human studies found that TNF-α G308A polymorphism, which caused a reduction of TNF-α function, could predict a subject’s resilience to sleep deprivation (Satterfield et al., 2015). This evidence together makes astrocyte an essential player in the normal sleeping process and a mediator of sleep homeostasis.

### Sleep Disturbance-Attenuated Microglial CX3C Chemokine Receptor 1 and P2Y12

Microglia serve as tissue-resident macrophages in the CNS and account for 5–12% of total brain cells (Lawson et al., 1990). They respond to disturbance of neural homeostasis caused by pathological challenges in the neural environment and are involved in the regulation of aging, neuropathic pain, and neurodegenerative disease (for review, Salter and Stevens (2017), Inoue and Tsuda (2018), Schwabe et al. (2020)). Although glial cells appear to be strong candidates of sleep homeostasis, the role microglia play in the process is still underestimated. Deurveilher et al. (2021) summarized the cell population, morphology, microglia genes, and physiology feature changes during daytime and sleep. They claimed that microglia cell intensity (number) was not changed after sleep loss. Recent research also showed the circadian rhythm of microglia is accompanied by morphological and molecular phenotypic changes (Deurveilher et al., 2021).

The fractalkine (CX3CL1) and its receptor CX3C chemokine receptor 1 (CX3CR1) and P2Y12, a chemoreceptor for adenosine diphosphate (ADP) that belongs to the Gi class of a group of G protein-coupled (GPCR) purinergic receptors, were found to be associated with sleep disturbance and were subjected to alteration following sleep deprivation. Microglial mRNA levels of CX3CR1 and P2Y12, two widely expressed microglial receptors in the CNS, were significantly reduced 72 h later following sleep deprivation in the hippocampus (Tuan and Lee, 2019). The increase in these mRNA was specifically in the brain region and highly relevant with age. In the medial prefrontal cortex, sleep deprivation resulted in less reduction of microglia gene expression in old mice compared with young mice (Guo et al., 2019). However, it is hard to conclude from this evidence that how microglia participate in the regulation of the normal sleeping state or the transition between sleep and awake states.

### Sleep Disturbance-Induced Microglial Ionized Calcium-Binding Adaptor Molecule 1 and Astrocytic Glial Fibrillary Acidic Protein

Microglia also participate in the adenosine signaling pathway regulating the sleep process. Chronic sleep deprivation increases the permeability of BBB and the expression of A2a receptor in multiple brain regions including hippocampus, basal nuclei, and cerebral cortex (Hurtado-Alvarado et al., 2016). Ionized calcium-binding adaptor molecule 1 (Iba-1), also known as Aif-1 (allograft inflammatory factor 1), and glial fibrillary acidic protein (GFAP) in microglia and astrocytes were induced by sleep deprivation. While the application of A2a-specific antagonist can attenuate the increase of iba-1 and GFAP induced by sleep deprivation (Hurtado-Alvarado et al., 2016). A2a receptor was shown to regulate the average capillary cerebral blood flow (CBF) of multiple cortical regions during rapid eye movement (REM) sleep, while there is no difference in capillary CBF between active awake and NREM sleep (van Calker et al., 2019; Tsai et al., 2021). These findings suggest that the microglia-adenosine interaction has a crucial role in sleep loss. However, it is still unclear whether microglia are directly involved in A2a receptor-mediated synaptic function in the regulation of sleep homeostasis. It is known that one of the major functions of microglia is phagocytosis. Relying on this function, microglia not only control synaptic punning (Tuan and Lee, 2019) but also clean up synaptic elements following overexcitation caused by sleep loss in the cortex and hippocampus (Bellesi et al., 2017; Tuan and Lee, 2019). Importantly, research conducted by Gentry et al. (2022) showed that microglia is essential for synaptic homeostasis and the protection of memories potentially through the upregulation of synaptic-homeostasis-related genes and further protection of nascent dendritic spines that may be removed during recovery sleep. Therefore, microglia are responsible for synaptic pruning not only during normal development but also when against pathological stimulation in the CNS (Ho, 2019; Sellgren et al., 2019). Lack of microglia-mediated synaptic pruning and clearance may result in sleep disturbance and further impairment of synaptic-plasticity-dependent higher-order cognitive function (Tuan and Lee, 2019; Deurveilher et al., 2021). Microglial CX3C-chemokine receptor 1 (CX3CR1) deficiency-attenuated neuroinflammation and the related synaptic pruning lead to cognition decline during sleep deprivation (Xin et al., 2021). Cytokine is another regulatory substance mediating the function of microglia in the sleep process (Qiu et al., 2021). It has been discovered that chronic deprivation of sleep drastically increases the expression of Iba-1 and glial fibrillary acidic protein (GFAP) levels which are receptively microglial- and astrocytic-specific markers (Bellesi et al., 2017).

### Microglial Proinflammatory Cytokine in Sleep Disturbance

Systematic proinflammatory cytokines including IL-6 and TNF-α could facilitate the NREM sleep, while anti-proinflammatory cytokines including IL-4 and IL-10 could generate the opposite effect (Rico-Rosillo and Vega-Robledo, 2018). However, the meta-analysis revealed the covariation of cytokines and sleep duration. Comparing short and long sleep duration together, sleep disturbance is associated with a significantly higher level of systematic IL-6 (Irwin et al., 2016). Although TNF-α is not remarkably associated with sleep duration (Irwin et al., 2016), chronic sleep disturbance induced by circadian misalignment could rise up the TNF-α and IL-10 levels in plasma (Wright et al., 2015). Short-term sleep loss in zebra finch remarkably increased the proinflammatory [interleukin (IL)-1b and IL-6] cytokine gene expression but reduced the anti-inflammatory (IL-10) cytokine gene expression in the CNS, specifically the hippocampus (Cooper et al., 2019). Sleep disturbances can increase inflammatory responses through the release of a series of inflammatory factors such as IL-1β, IL-6, and TNF-α, which further exacerbates symptoms or the risk of neurodegenerative diseases (Marshall and Born, 2002; Weil et al., 2009; Besedovsky et al., 2019; Green et al., 2020). Observation and findings concerning microglia function implicate an indispensable role of microglial immune response in sleep regulation. Interestingly, as elaborated in the above section, TNF-α in microglia plays an awaking-favor role suggesting that microglia could regulate the sleeping loss through shared signaling pathways.

## Astrocytic and Microglial Roles in Alzheimer’s Disease

### Astrocytic and Microglial Aβ and Tau in Alzheimer’s Disease

Astrocytes and microglia are the major players of innate immune response but they contribute to the pathology of AD in different manners. Aβ and tau are two major substances of AD; astrocytes and microglia contribute to their clearance and halt their spreading (Fagan and Holtzman, 2000; Gratuze et al., 2021; Mahan et al., 2022). Astrocytes in the healthy brain remain in resting state. Accumulation of Aβ and NFT in the CNS triggers the activation of astrocytes before the onset of AD psychosis, which will further induce the release of a series of proinflammatory factors and ultimately neuroinflammation (Berridge, 2014). In turn, the neuroinflammation further accelerates the progress of AD. Astrocytes are one of the important resources of adenosine in the CNS. Astrocytes dysfunction in LH could thus be responsible for the sleep disturbance.

### Astrocytic Aβ Interaction in Alzheimer’s Disease

At the early stage of AD, astrocytes serve as a protector of the neuro system to digest accumulated Aβ and transport it out of the BBB with the facilitation of chaperones, one type of heat shock proteins (Ries and Sastre, 2016). While along with the surge of Aβ and formation of NFT, overactivated astrocytes introduce perturbation to the CNS and cause damage to the surrounding neurons which results in the imbalance of immune-neuron homeostasis (Agostinho et al., 2010; Avila-Muñoz and Arias, 2014; Ahmad et al., 2019) and Aβ accumulation in both neurons and astrocytes. A previous study revealed that the entorhinal cortex of patients with AD exhibited an increased level of Aβ, suggesting that astrocyte was also a victim of Aβ accumulation in AD generation.

### Astrocytic Interaction With Aβ in Central Nervous System

How do astrocytes interact with Aβ in CNS during the onset and progression of AD? Conventionally, it is believed that as CNS immune cells, astrocytes eliminate and degrade Aβ through proteolysis, which involves a variety of proteases including neprilysin, endothelin-converting enzyme, insulin-degrading enzyme, and matrix metalloproteases (Apelt et al., 2003; Ahmad et al., 2019). Deficiency in these proteins directly leads to abnormal degradation and accumulation of Aβ. Disruption of the sleep-awake cycle and decrease in sleep duration directly impair the ability of astrocytes in Aβ clearance (Sunkaria and Bhardwaj, 2022).

### Astrocytic Apolipoprotein E-Induced Aβ Clearance in Alzheimer’s Disease

ApoE, a lipid-loaded protein that facilitates the transportation of Aβ by binding with ApoE receptors on the surface of the cell membrane, also mediates the astrocyte and Aβ interaction (Liao et al., 2017; Zhao et al., 2018; Uddin et al., 2019). Among the ApoE family, ApoE2 is considered as a neuroprotector against AD pathology, while ApoE4 promotes the Aβ accumulation and AD symptoms (Fleisher et al., 2013; Serrano-Pozo et al., 2015). The major source of ApoE is reported to be glial cells (Mahley, 2016). To form ApoE particles, they will be lapidated by ATP-binding cassette A1 or G1 transporters. These particles are responsible for the transportation of lipids in the CNS. Besides, ApoE is essential for the homeostasis of lipid metabolism (Farmer et al., 2019) and endocytic clearance of Aβ in astrocytes (Prasad and Rao, 2018). Acidification of endosome in ApoE4 astrocytes increases the expression of low-density lipoprotein receptor-related protein in intracellular compartments, which is responsible for the deficit of Aβ clearance through endocytosis (Verghese et al., 2013; Prasad and Rao, 2018). Therefore, astrocytic ApoE might serve as the bridge in the interaction between astrocyte and Aβ in the exploration of AD pathology.

### Microglial Aβ Clearance and Aggregation in Alzheimer’s Disease

**FIGURE 1:**
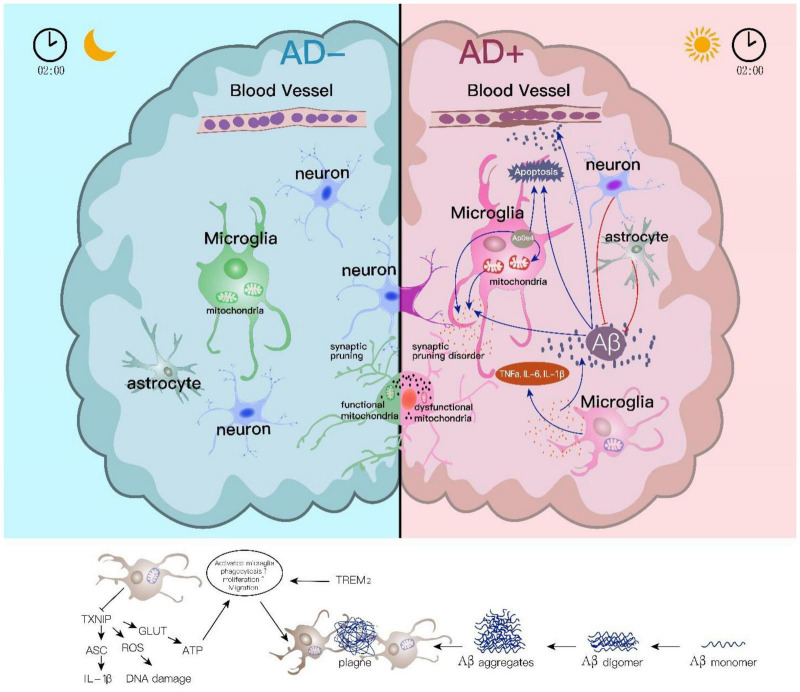
Astrocyte and microglial roles in sleep disturbance linked Alzheimer’s disease. Sleep and CNS immune influence each other. Microglial responses in the sleep/wake are essential for Aβ clearance and inflammatory activation. Aβ accumulation induces abnormal mitochondrial function in microglia which further activates the release of inflammatory cytokines, while Aβ clearance by microglia phagocytosis relies on the stimulation of Aβ itself. TREM2 in the microglia membrane combines with Aβ and then enhances its ability on Aβ phagocytosis. The downregulated TXNIP induces ROS inhibition and further causes DNA damage during sleep disorders. On the contrary, lower TXNIP under sleep disturbances could increase NLRP3 inflammasome activation and IL-1β-initiated inflammatory response. Apart from the role in Aβ clearance and Aβ involved inflammation, microglia directly participate in synapse removal or “synaptic stripping,” and this is regulated by normal sleep/wake rhythm.

The function of microglia in AD is partially overlapped with astrocytes carrying distinct features. Microglia also gather around Aβ plaques in the CNS of patients with AD (Perlmutter et al., 1992; Rozemuller et al., 1992). Microglia-mediated Aβ clearance relies on the stimulation of Aβ itself. TREM2, expressed in the microglia membrane and its exosome membrane, combines with Aβ and then changes its surrounding inflammatory microenvironment further promoting microglia phagocytosis of Aβ (Huang et al., 2022). The complex pathological changes of AD, including pTau, Aβ, and pSyn, also affect the microglia phenotype in turn (Fixemer et al., 2022). Aβ causes dysregulation of mitochondria in microglia, which further activates them to trigger phagocytosis activity and, in the meanwhile, stimulates the release of proinflammatory cytokines to remove extracellular Aβ plaques (Chiozzi et al., 2019). Recently, Oualid Sbai’s team showed that RAGE-TXNIP axis inhibition in microglia could reduce Aβ transport from the cell surface to mitochondria and restore mitochondrial function and Aβ toxicity, which in turn inhibit NLRP3 inflammasome activation (Sbai et al., 2022). Disruption of the sleep-awake cycle and decrease in sleep duration directly impair the ability of microglia in Aβ clearance (as shown in Figure 1).

### Proinflammation-Induced Microglial Aβ Aggregation in Alzheimer’s Disease

Proinflammatory cytokines, including IL-1b, IL6, and TNF-alpha, are elevated in cultured neurons derived from patients with AD (Murphy et al., 1998; Liu and Hong, 2003; Ye et al., 2013; Wood et al., 2015; Barroeta-Espar et al., 2019). These proinflammatory cytokines could derive from microglia upon being stimulated by extracellular accumulated Aβ. Microglia participate in soluble Aβ (sAβ) macrophages *in vitro* and *in vivo*, which have been shown to be trafficked into the late lysosomal compartment and degraded (Prokop et al., 2013; John and Reddy, 2021). In contrast, some of the proinflammatory and anti-inflammatory cytokines that mediate Aβ clearance by astrocytes are also the major ones that microglia secrete to promote Aβ aggregation (Decourt et al., 2017; Ng et al., 2018; Taipa et al., 2019), suggesting a reciprocal interaction between Aβ aggregation and microglia dominating clearance.

### Microglial Accumulated Tau in Alzheimer’s Disease

Although Aβ is considered as the driving force of tau pathology, downstream biological pathways mediated by Aβ and tau in AD could be unrelated to each other (van der Kant et al., 2020). Tau accumulates in the entorhinal cortex during the onset of AD and propagates to the neocortex along with the development of the disease (van der Kant et al., 2020). Microglia facilitate the propagation of tau among neurons through exosome secretion from the entorhinal cortex to the hippocampus (Asai et al., 2015; Hopp et al., 2018).

### Microglial Apolipoprotein E and Triggering Receptor Expressed on Myeloid Cells 2 in Alzheimer’s Disease

Although the leading position between tauopathy and microglia activation is undefined, there is no doubt that tauopathy is not only the cause but also the consequence of microglia activation in AD (for review, Vogels et al., 2019). Among those AD risk genes, ApoE and triggering receptor expressed on myeloid cells 2 (TREM2) are the main regulators of lipid metabolism by glial cells. ApoE is predominantly expressed in astrocytes, while its low expression in microglia under normal stage increases significantly close to astrocyte-level when reactive (Wang et al., 2021; Parhizkar and Holtzman, 2022). TREM2 is expressed in microglia with higher specificity (Pfrieger, 2003; Kim et al., 2009). Those microglia expressing AD risk genes including ApoE and TREM2 are classified as disease-associated microglia (Kfoury et al., 2012), which are the main players among the microglia family in the participation of AD. Alteration of TREM2 changes the homeostasis of microglia. For example, overexpression of TREM2 upregulates the homeostatic genes in microglia (Jiang et al., 2016). In contrast, downregulating TREM2 results in failed activation of microglia and release of proinflammatory factors that usually favor the formation of NFT (Jiang et al., 2018). Therefore, ApoE and TREM2 might be more important in controlling the progress of AD pathology, rather than timing the onset of it.

## Astrocytic and Microglial Roles in Alzheimer’s Disease and Sleep

### Sleep Disturbance in Alzheimer’s Disease

The major pathological features of AD include the accumulation of Aβ plaques and the neurofibrillary tangles (NFTs), which resulted from hyperphosphorylated tau protein. These substances cause oxidative stress, cell death, and destabilizing microtubules in the CNS, which triggers inflammatory cascades and causes physiological and cognitive deficits (McNaull et al., 2010; Heneka et al., 2015; Bronzuoli et al., 2016; Calsolaro and Edison, 2016; Guo et al., 2016). Poor sleep widely occurs during the normal aging process. However, sleep disturbance in AD is very common which adds extra burden and stress on patients. A previous study assessed the sleep quality of 215 patients with AD and found that 24.5% of them showed sleep disturbance with different magnitudes varying from mild to medium level (Moran et al., 2005). AD and sleep disturbance mutually affect each other. Neurodegeneration and immune reaction of AD may disrupt the sleep-wake cycle (Vanderheyden et al., 2018b). In the meanwhile, sleep disturbance further accelerates the pathological progress and symptoms of AD including impairment of cognitive function (Rauchs et al., 2008; Ooms et al., 2014). Therefore, characterizing the pathological mechanism linking sleep disturbance and AD will provide new insights on developing more potent therapeutic treatments.

### Aβ and Tau in Alzheimer’s Disease and Sleep Disturbance

It is thus reasonable to speculate that Aβ and tau triggering CNS immune reaction may play a noticeable role in the sleeping disturbance seen in AD. Accumulated Aβ would further trigger more severe synaptic inhibition and cause more synapse loss in the sleep-associated neural circuit to deteriorate the sleeping disturbance in AD (Spinedi and Cardinali, 2019). This evidence implicates that a direct interaction between Aβ and synapse might link sleep regulation and AD, which provide a novel target for treating sleeping disturbance in AD. In fact, loss of sleep is sufficient to promote the accumulation of Aβ in the CNS in drosophila, rodents, and human (Kang et al., 2009; Tabuchi et al., 2015; Shokri-Kojori et al., 2018). Moreover, Aβ-induced synaptic deficits are major reasons driving AD pathogenesis (Hardy and Selkoe, 2002; Forloni and Balducci, 2018; Torres et al., 2021). Direct application of Aβ oligomer could also lead to long-term depression in synapses and cognitive deficits (Shankar et al., 2008; Marcello et al., 2019; Yu et al., 2021). Therefore, it is highly possible that Aβ accumulation in AD could impact synaptic function in neural circuits associated with sleep, followed by sleep disturbance and poor performance of cognition. However, how direct application of adenosine may regulate AB accumulation in AD and how it may change the sleep-awake cycle of patients with AD still need further clarification.

### Astrocytic Apolipoprotein E in Sleep and Alzheimer’s Disease

Astrocytic gene ApoE might be one of the factors causing sleep disturbance in AD. The increase of ApoE expression has been seen in the CNS of AD, which is considered to enhance the amyloid pathology (Muñoz et al., 2019). Patients with AD carrying homozygous ApoEε4 gene are found to coexist with sleep disorders at a higher ratio (Koo et al., 2019; Pyun et al., 2019). However, ApoE facilitates the transportation of Aβ by binding with ApoE receptors on the surface of the cell membrane (Liao et al., 2017; Zhao et al., 2018; Uddin et al., 2019). The increased Aβ may promote the degradation of CNS and the change of circadian gene expression, thus interfering with the behavioral regulatory circuit and leading to Sundown syndrome. This also resulted in a higher proportion of patients with AD with homozygous ApoEε4 gene coexisting with sleep disorders (Koo et al., 2019; Pyun et al., 2019). We speculate that increased ApoE in AD patients with sundown syndrome was a protective mechanism against Aβ accumulation. In fact, delirium occurrence or even exacerbation during the evening in AD is consistent with “sundowning” (Vitiello et al., 1992; Volicer et al., 2001), and the effectiveness of circadian alignment by bright light therapy and melatonin contradicts the sundowning and other sleep-wake disorders in patients with AD (Cardinali et al., 2010; Sharma et al., 2021). The fluctuation curve of ApoE in astrocytes across different sleep stages remains unclear.

### Microglial Proinflammatory Status in Alzheimer’s Disease and Sleep Disturbance

Microglia are the major source of cytokine in the CNS. Microglia also participate in sleep loss by enhancing the TNF-α signal. Interestingly, in patients with AD, proinflammatory cytokines, including IL-1b, IL6, and TNF-α, are particularly elevated through Aβ stimulating microglia (Murphy et al., 1998; Liu and Hong, 2003; Ye et al., 2013; Wood et al., 2015; Barroeta-Espar et al., 2019) and disrupt the homeostasis of immune system in the CNS, which may cause direct result in sleep disturbance seen in patients with AD. Therefore, microglia-mediated proinflammatory cytokines including TNF-α may underly the sleep disturbance seen in AD.

## Conclusion and Future Directions

**FIGURE 2:**
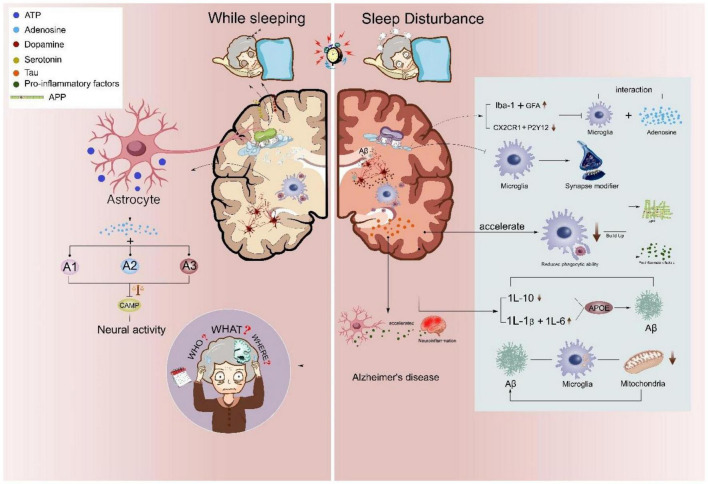
Astrocyte and microglial roles in sleep disturbance linked Alzheimer’s disease. Sleep disturbances accelerate the neuropathological changes of AD. During normal sleep/wake rhythm, astrocytic adenosinergic A1, A2, and A3 inhibit neural overactivation, while sleep disturbance attenuates microglial CX3CR1 and P2Y12 further inhibiting the phagocytic capacity of microglia. Abnormal sleep rhythms also promote microglial Iba-1 and astrocytic glial fibrillary acidic protein (GFAP) and increase microglia-mediated proinflammatory releases, such as IL-1b, IL-6, IL-10, and TNFα. Activated inflammatory status further induces microglial Aβ aggregation and microglial-accumulated tau in AD.

Loss of sleep not only deteriorates AD progress by emotionally affecting the mental states of patients but also accelerates the pathological changes of AD. Sleep disorders and AD partially share the common pathomechanisms with similar cognitive dysfunction. Therefore, it is important to fully characterize these pathological changes for better therapeutic intervention. In this review, we navigated through the mechanism associated with astrocytes and microglia in both sleep and AD (as shown in Figure 2). We particularly summarized substrates shared by astrocytes and microglia, including proinflammatory and anti-inflammatory factors (IL6, TNFα, IL-1β, and IL10). In the meanwhile, the discrepancy was presented between microglia and astrocytes in AD, for example, the specificity of ApoE and TREM. Aβ appears to be a strong candidate in linking sleep disturbance through the above-mentioned cytokines and genes with AD risk involving the engagement of both astrocytes and microglia. In future, certain questions still remain to be addressed in the field of exploring joint mechanisms underlying sleep disturbance and AD. (1) The fluctuation curve of ApoE in astrocytes across different sleep stages remains unclear. (2) How the direct application of adenosine may regulate AB accumulation in AD and how it may change the sleep-awake cycle of patients with AD still await further clarification. Comprehensive studies are needed to better answer these questions and potentially inspire better ideas for therapeutic treatments of sleep disturbance and AD. (3) The interaction between Aβ and synapse in simultaneously accelerating sleep disorder and AD provides a novel point to study the joint mechanism underlying these two disorders. However, the details of neural mechanisms at synaptic, cellular, and circuitry levels require more exploration in the future.

## Author Contributions

S-YX and Y-JL wrote the main body of the manuscript. WL, CX, and S-DL did the proofreading and grammar checking. WL and Z-WS contributed to the graph abstract drawing. S-YX, Z-HY, and S-DL designed the study and guided the writing. Y-JL, Z-HY, and S-DL contributed to the manuscript revision. All authors contributed to the article and approved the submitted version.

## Conflict of Interest

The authors declare that the research was conducted in the absence of any commercial or financial relationships that could be construed as a potential conflict of interest.

## Publisher’s Note

All claims expressed in this article are solely those of the authors and do not necessarily represent those of their affiliated organizations, or those of the publisher, the editors and the reviewers. Any product that may be evaluated in this article, or claim that may be made by its manufacturer, is not guaranteed or endorsed by the publisher.

## Abbreviations

EEG: electroencephalogram
NREM: non-rapid eye movement
LH: lateral hypothalamus
BBB: blood–brain barrier
AD: Alzheimer’s disease
ApoE: apolipoprotein
PSEN1: presenilin 1
PSEN2: presenilin 2
A β: amyloid beta
ATP: adenosine triphosphate
cAMP: cyclic adenosine monophosphate
TNF α: tumor necrosis factor alpha
GFAP: glial fibrillary acidic protein
IL: interleukin
NFTs: neurofibrillary tangles
TREM2: triggering receptor expressed on myeloid cells 2.
